# The myelin water imaging transcriptome: myelin water fraction regionally varies with oligodendrocyte-specific gene expression

**DOI:** 10.1186/s13041-024-01115-4

**Published:** 2024-07-23

**Authors:** Jaimie J. Lee, Paulina S. Scheuren, Hanwen Liu, Ryan W. J. Loke, Cornelia Laule, Catrina M. Loucks, John L.K. Kramer

**Affiliations:** 1 International Collaboration on Repair Discoveries (ICORD), University of British Columbia, Vancouver, BC Canada; 2 Department of Anesthesiology, Pharmacology, and Therapeutics, University of British Columbia, Vancouver, BC Canada; 3 Department of Medicine, University of British Columbia, Vancouver, BC Canada; 4 Department of Radiology, University of British Columbia, Vancouver, BC Canada; 5 Department of Pathology and Laboratory Medicine, University of British Columbia, Vancouver, BC Canada; 6 Department of Physics and Astronomy, University of British Columbia, Vancouver, BC Canada; 7 Division of Translational Therapeutics, Department of Pediatrics, University of British Columbia, Vancouver, BC Canada; 8 BC Children’s Hospital Research Institute, Vancouver, BC Canada

**Keywords:** Myelin, Neuroimaging transcriptomics, Transcriptome, Myelin water fraction, Myelin water imaging, Gene expression, White matter

## Abstract

Identifying sensitive and specific measures that can quantify myelin are instrumental in characterizing microstructural changes in neurological conditions. Neuroimaging transcriptomics is emerging as a valuable technique in this regard, offering insights into the molecular basis of promising candidates for myelin quantification, such as myelin water fraction (MWF). We aimed to demonstrate the utility of neuroimaging transcriptomics by validating MWF as a myelin measure. We utilized data from a normative MWF brain atlas, comprised of 50 healthy subjects (mean age = 25 years, range = 17–42 years) scanned at 3 Tesla. Magnetic resonance imaging data included myelin water imaging to extract MWF and T1 anatomical scans for image registration and segmentation. We investigated the inter-regional distributions of gene expression data from the Allen Human Brain Atlas in conjunction with inter-regional MWF distribution patterns. Pearson correlations were used to identify genes with expression profiles mirroring MWF. The Single Cell Type Atlas from the Human Protein Atlas was leveraged to classify genes into gene sets with high cell type specificity, and a control gene set with low cell type specificity. Then, we compared the Pearson correlation coefficients for each gene set to determine if cell type-specific gene expression signatures correlate with MWF. Pearson correlation coefficients between MWF and gene expression for oligodendrocytes and adipocytes were significantly higher than for the control gene set, whereas correlations between MWF and inhibitory/excitatory neurons were significantly lower. Our approach in integrating transcriptomics with neuroimaging measures supports an emerging technique for understanding and validating MRI-derived markers such as MWF.

**Supplementary Information:**

The online version contains supplementary material available at 10.1186/s13041-024-01115-4.

## Introduction

Myelin facilitates the rapid propagation of action potentials in the central nervous system. Pathological changes in myelin are associated with numerous disease states, including multiple sclerosis, giving rise to the need for objective quantification markers [1, 2]. To this end, a variety of “myelin” neuroimaging measures have been proposed [3–9].

Myelin water fraction (MWF) has emerged as a promising magnetic resonance imaging (MRI) metric for assessing demyelination in multiple sclerosis pathology. MWF quantifies water trapped between myelin lipid bilayers relative to water in other spaces. The validation of MWF as a specific and sensitive marker for myelin is supported by post-mortem studies with histological measures of myelin density [10–12]. A recent neuroimaging transcriptomics study in young men has also revealed that MWF determined using one particular type of data acquisition method is associated with myelin gene expression in predominantly gray matter regions across the cerebral cortex [13].

Neuroimaging transcriptomics is a new and emerging field of research that combines transcriptomics with neuroimaging to gain insights into the cellular and molecular underpinnings of brain function and anatomy [14]. The basic premise of the approach is that genes with expression patterns matching a neuroimaging feature represent the molecular signature of that feature. In healthy individuals, MWF varies significantly across white matter brain regions [15], which should, in theory, correspond with the expression pattern of oligodendrocyte-associated genes, given the key role of oligodendrocytes in myelin production and maintenance.

The aim of the current study was to examine the spatial relationship between MWF and gene expression in white matter tracts in the healthy adult human brain. We hypothesized that regional variations in MWF will be positively and uniquely associated with myelin-related gene expression.

## Results and discussion

**Fig. 1:**
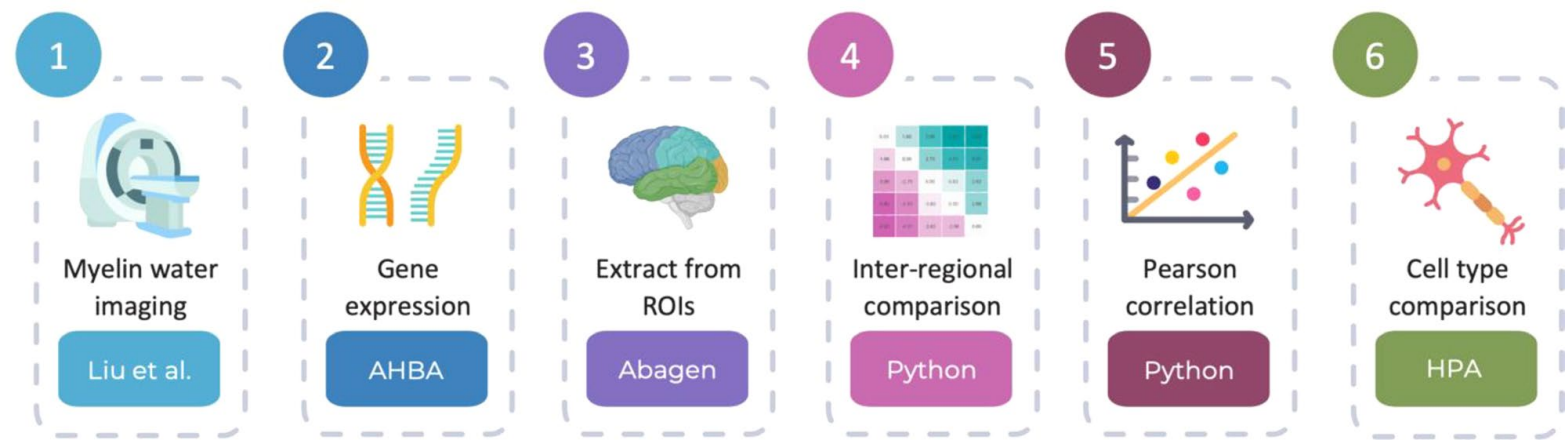
Overview of methodological pipeline from myelin water imaging to cell type comparison. Step 1 obtains myelin water imaging data from Liu et al. (2019) [15]. Step 2 retrieves gene expression data from the Allen Human Brain Atlas (AHBA). Step 3 extracts myelin water fraction (MWF) and gene expression data from regions of interest (ROIs) using the Abagen toolbox. Step 4 generates inter-regional pairwise comparison matrices for MWF and gene expression. In these matrices, each cell represents the Cohen’s d effect size between two ROIs. Step 5 correlates MWF with gene expression pairwise comparison matrices using Pearson correlation. Finally, Step 6 compares cell type-specific data referencing the Human Protein Atlas (HPA). Select images in this figure were sourced from BioRender and Canva [16–20]

**Fig. 2:**
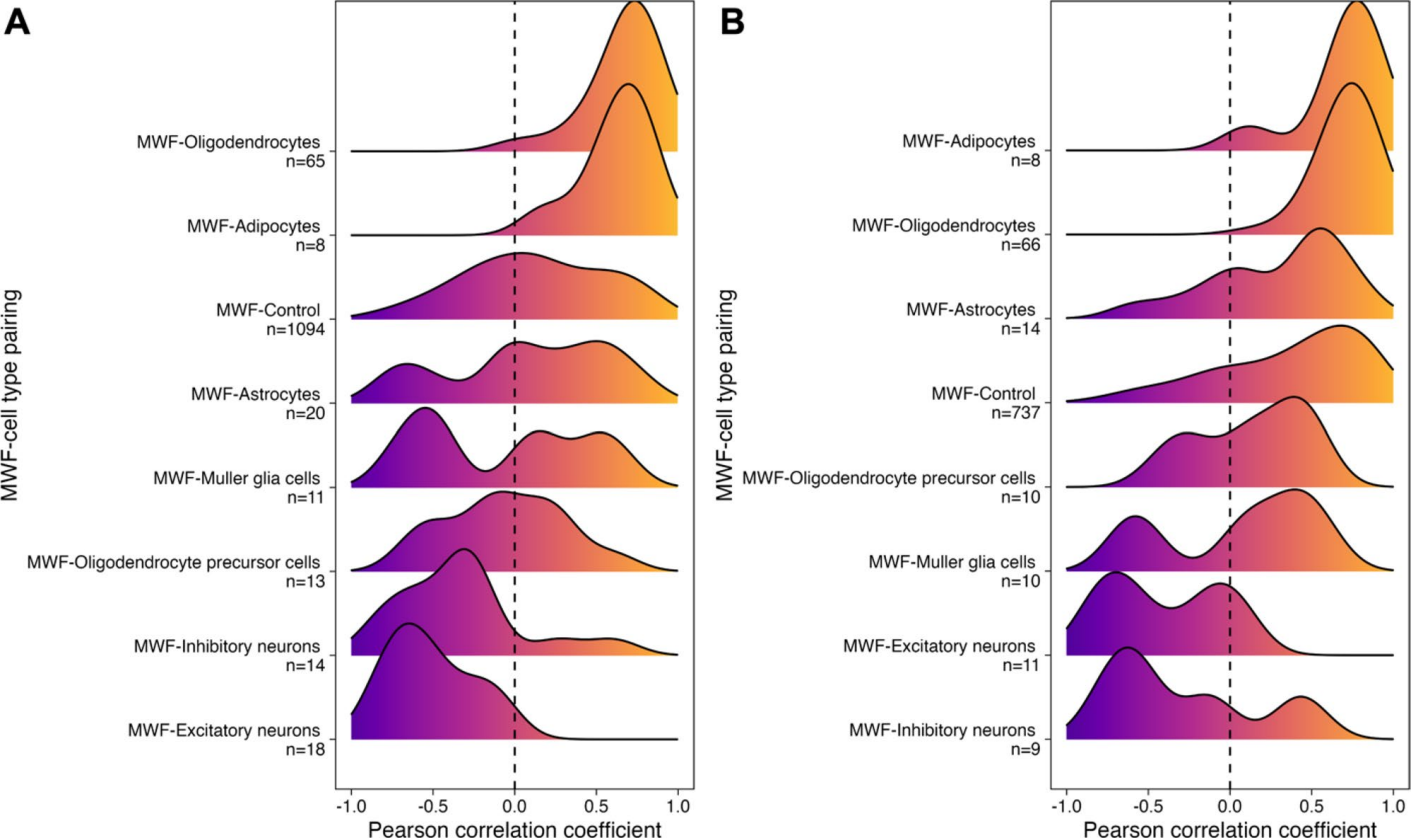
Distribution of Pearson correlation coefficients across MWF-cell type pairings at 2 mm (**A**) and 0 mm parcellation thresholds (**B**). The ridgeline plots depict the distribution of Pearson correlation coefficients for each MWF-cell type pairing, indicating the strength and direction of the correlation between MWF and gene expression. The number of genes in each gene set is denoted by “n”. The dashed line represents the zero-correlation mark, where values to the left suggest a negative correlation and values to the right indicate a positive correlation. Cell types range from neurons to glial cells, with a control group (low cell type specificity) included for reference. Genes that were classified as having low cell type specificity in the Human Protein Atlas were present in roughly similar levels across all cell types

An overview of the methodological pipeline is illustrated in Fig. 1. Of the 11,074 genes initially included in our analysis, 1,257 matched to a Human Protein Atlas brain “cell type-enriched” or “low cell type specificity” gene set. The mean MWF-low cell type specificity gene set pairing (r-value) was 0.13, with a standard deviation of ± 0.43 (*n* = 1,094 genes). Seven cell type-enriched gene sets, with *n* > 5 genes, were included in our final statistical analysis: adipocytes (*n* = 8 genes), Muller glial cells (*n* = 11 genes), oligodendrocyte precursor cells (*n* = 13 genes), inhibitory neurons (*n* = 14 genes), excitatory neurons (*n* = 18 genes), astrocytes (*n* = 20 genes), and oligodendrocytes (*n* = 65 genes). Non-parametric statistical testing revealed significant differences across gene sets (Fig. 2A; Kruskal-Wallis: test statistic = 155.0, df = 7, *p* < 0.05). Compared to a low cell type specificity gene set (control; *n* = 1,094), MWF-gene set pairings for oligodendrocytes and adipocytes were significantly higher, while inhibitory and excitatory neurons were significantly lower (Table 1). The MWF-gene set pairings for oligodendrocytes and adipocytes were also significantly higher compared to astrocytes, Muller glial cells, and oligodendrocyte precursor cells (Supplementary Table 1). There were no significant differences between MWF-astrocytes, MWF-Muller glial cells, and MWF-oligodendrocyte precursor cell gene set pairings and the MWF-low cell type specificity (control) gene set pairing.

**Table 1:** Comparison of RNA single cell types to a control group. Each row evaluates whether MWF-gene pairings in high cell type specificity genes sets differ from the MWF-control gene set pairing, using the Mann-Whitney U statistic. The control set consisted of genes identified as having low cell type specificity in the Human Protein Atlas and were present in roughly similar levels across all cell types. Adjustments for multiple comparisons were made using the Bonferroni correction method. *: *p* < 0.05, **: *p* < 0.01, ***: *p* < 0.001, ****: *p* < 0.0001, *****: p is approximating 0

High Cell Type Specificity Gene Set	Test Statistic	Std. Error	Std. Test Statistic	*P* value	Adjusted *p* value
Oligodendrocytes	439.48	45.83	9.59	0.00 *****	0.00 *****
Excitatory neurons	-472.61	85.30	-5.54	3.02E-8 ****	8.45E-7 ****
Inhibitory neurons	-350.88	96.55	-3.63	2.79E-4 ***	7.81E-3**
Oligodendrocyte precursor cells	-152.15	100.15	-1.52	0.13	1.00
Muller glial cells	-138.11	108.78	-1.27	0.20	1.00
Astrocytes	-23.18	81.00	-0.29	0.77	1.00
Adipocytes	406.62	127.38	3.19	1.41E-3 **	3.95E-2 *

Aligning with our hypothesis, the MWF-oligodendrocyte gene set pairing was: (a) positive and (b) significantly higher than the pairing for control genes (i.e., the low cell type specificity gene set), in addition to all other gene set pairings. The opposite was the case for MWF-inhibitory and MWF-excitatory neuron gene set pairings, which were: (a) negative and (b) significantly lower than the pairing for control genes, suggesting that areas with higher gray matter content are associated with lower MWF. MWF-astrocyte, MWF-Muller glial cell, and MWF-oligodendrocyte precursor cell gene pairings were no different than the pairing for the control gene set. Interestingly, the MWF-adipocyte gene set pairing was also significantly higher than the control pairing – a novel observation that may reflect adipocytes as the main constituent of the myelin lipid bilayer [21, 22]. A sensitivity analysis performed at 0 mm sample-to-region matching tolerance (i.e., all microarray samples derived strictly from within the region of interest; Supplementary Fig. 2) confirmed our primary observations, with oligodendrocytes and adipocytes ranked the most strongly correlated with MWF (Fig. 2B; Table 1). Genes that were of low cell type specificity, as well as astrocytes, increased their correlation with MWF at 0 mm compared to 2 mm (Supplementary Table 2). However, low cell type specificity and astrocyte genes were still commonly negatively associated with MWF, which was rarely the case for oligodendrocytes or adipocytes.

To the best of our knowledge, we are the first to have employed a neuroimaging transcriptomics approach to understand spatial variations in MWF in white matter tracts. One earlier study examined mixed white and gray matter regions [14], where it is possible that associations between gene expression and MWF could reflect, to a large degree, differences in gray matter composition, where myelin levels are ~ 10x lower than white matter. The focus on mixed gray and white matter regions may also explain the significant negative MWF-astrocyte gene set pairing in another previous study [13]. Moreover, substantial differences across white matter tracts have been previously reported for MWF, with nearly a two-fold difference between the internal capsule and the corpus collosum [15]. Based on our results, these MWF variations reflect normal phenotypic variations in white matter gene expression in the brain. Such conclusion can be drawn from the association between MWF and gene expression patterns of cells responsible for forming white matter structures, specifically oligodendrocytes and adipocytes.

In addition to examining mixed white and gray matter regions, previous neuroimaging transcriptomics studies have employed different methods of estimating MWF. The combined gradient and spin-echo (GRASE) acquisition method used in the current study is a well-established myelin water imaging approach, having been extensively validated as an objective marker of myelin in the human brain [23, 24]. Conversely, the multicomponent driven equilibrium single pulse observation of T1 and T2 (mcDESPOT) acquisition method is a newer approach, whose primary advantage is faster acquisition time and higher signal to noise ratio [25]. However, as noted by West and others [13, 26–28], the conventional mcDESPOT fitting scheme may lead to an inherent and unpredictable bias in MWF estimation. Additionally, in the context of neuroimaging transcriptomics, it appears that mcDESPOT may produce less pronounced regional variations in MWF [29]. While regional differences across white matter tracts are typically more distinct with GRASE, suggesting more variability, it seems unlikely that the MWF derived from GRASE and mcDESPOT would yield comparable results. In this regard, demonstrating an association between GRASE-derived MWF and myelin related genes (i.e., oligodendrocytes) represents important and necessary validation of MWF as a myelin measure using neuroimaging transcriptomics.

Improved acquisition of mcDESPOT, namely BMC-mcDESPOT, demonstrates greater inter-regional MWF variations [30], more similar to those observed with GRASE. Additionally, while mcDESPOT tends to report higher MWF values than GRASE, we recognize this does not necessarily imply that GRASE is more accurate. MWF is influenced by various physiological factors and pulse sequence parameters that may introduce variability to the myelin measure [28, 31–33]. We also emphasize the importance of further histological validation of existing MRI-derived myelin measures. Specifically, validation across different age groups and use of fresh samples to avoid the biases associated with formalin fixation, which can influence MWF measurements and other nuclear magnetic resonance parameters [34, 35], may be worth investigating.

Our observations are also important in that MWF was estimated in both men and women, whereas Patel and colleagues only sampled MWF from males [13]. Methodologically, we incorporated a novel neuroimaging transcriptomics approach, characterizing regional variations in gene expression and MWF across white matter tracts as Cohen’s d effect sizes before estimating their spatial relationship. This was primarily done to further account for donor-level differences in regional gene expression that are often overlooked in neuroimaging transcriptomics studies. Further validation of this approach, including a direct comparison with other neuroimaging transcriptomics methods using age-matched cohorts from whom imaging and transcriptomics data are sourced, is warranted.

## Materials and methods

### MRI data acquisition

**Fig. 3:**
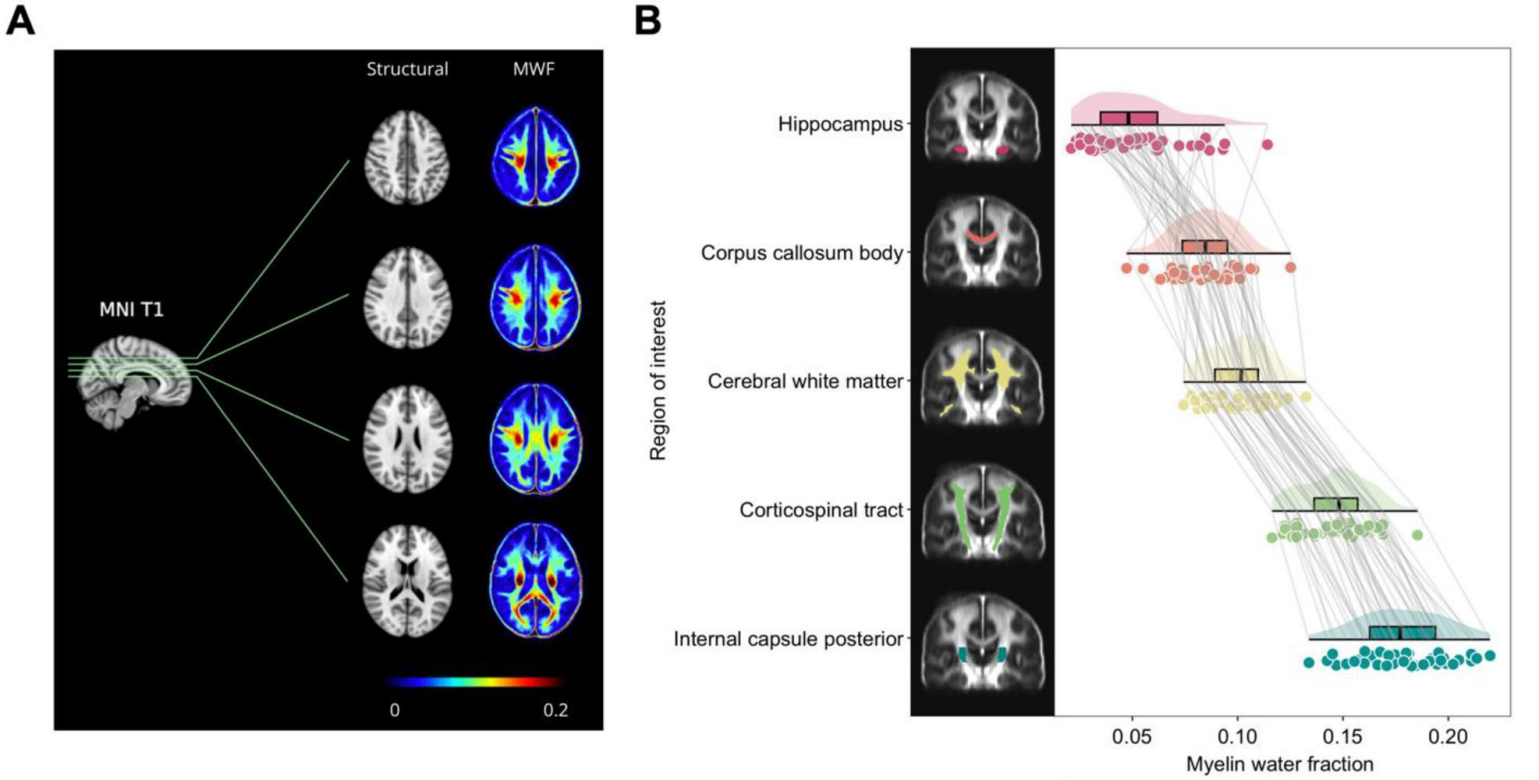
Myelin water imaging brain atlas. **A.** Structural T1 in MNI152 standard space (grayscale) and corresponding myelin water fraction (MWF) maps (colour scale: 0-0.2) across 50 healthy participants (adapted from Fig. 2. Liu, H., et al. [15]). **B.** Regional distribution of MWF across five brain regions of interest (ROIs). The anatomical location of the ROI masks in coronal view are accompanied by corresponding raincloud plots (violin plot, boxplot, and jittered data points). Gray lines connect MWF values across ROIs for individual participants

MRI data from a myelin water imaging brain atlas was used in our study [15] (Fig. 3A). The atlas comprises 50 healthy participants (25 males/25 females, mean age = 25 years, age range = 17–38 years) scanned at 3T using an 8-channel phased-array head coil (Achieva, Philips, Best, The Netherlands). Data included myelin water imaging scanned with a GRASE sequence (32-echo, TE/ΔTE/TR = 10/10/1000 ms, slices = 40, resolution = 1 × 1 × 2.5 mm^3^) and a 3DT1 whole brain turbo field echo (flip angle = 6°, TE/TR = 3.7/7.4 ms, slices = 160, resolution = 1 × 1 × 1 mm^3^).

### MWF calculation

For the MWF calculation, voxel-wise T2 distributions were calculated using non-negative least squares with stimulated echo correction [36]. MWF was defined as the fractional signal with T2 < 40 ms. Each participant’s MWF map was first registered to their corresponding 3DT1 space and subsequently warped to the standard 1 mm MNI152 space. MWF for each subject was extracted from five ROIs parcellated from the Johns Hopkins University atlas [37]. This included four white matter regions (i.e., internal capsule posterior, corticospinal tract, cerebral white matter, and corpus callosum body) and one predominantly gray matter region (hippocampus) (Fig. 3B).

### Transcriptomic data processing

The Allen Human Brain Atlas (AHBA) is an anatomically comprehensive transcriptional map quantifying gene expression levels from six healthy post-mortem whole brains (5 males/1 female, mean age = 42 years, age range = 24–57 years) [38].

The AHBA microarray dataset was preprocessed using the Abagen toolbox [39–41]. Regional microarray expression data were extracted from the same five ROIs as for MWF from each brain donor. Supplementary Fig. 1 illustrates MNI coordinates of each microarray probe included in our analysis. Microarray probes were filtered based on their expression intensity relative to background noise, such that probes with intensity less than the background in > = 50% of samples across donors were discarded. When multiple probes indexed the expression of the same gene in an ROI, we selected the probe with the highest average correlation to other probes across all samples (i.e., correlation intensity). Samples were assigned to a brain region if their MNI coordinates were within 2 mm of each parcellation. A sensitivity analysis was also performed at 0 mm (Supplementary Fig. 2, Fig. 2B). The MNI coordinates of tissue samples were updated to those generated via non-linear registration using Advanced Normalization Tools (ANTs) [42]. Normalization of tissue sample expression values across genes using a scaled robust sigmoid function was conducted to account for individual differences in donor brain expression [43].

To ensure the representativeness of the spatial variations in gene expression across donors, we only examined genes that passed the following two conditions in our downstream analyses. First, we only retained genes with expression data from all six AHBA brain donors in each ROI to maximize the number of microarray probes sampled per region. Second, we completed a pairwise Pearson correlation analysis between donors and only retained genes with a donor-to-median correlation coefficient *r* > 0.4, a threshold indicative of a moderately consistent expression pattern across donors [13]. A total of 11,074 genes passed the two-stage consistency filter and were used in our downstream analyses.

### Neuroimaging transcriptomics analysis

**Fig. 4:**
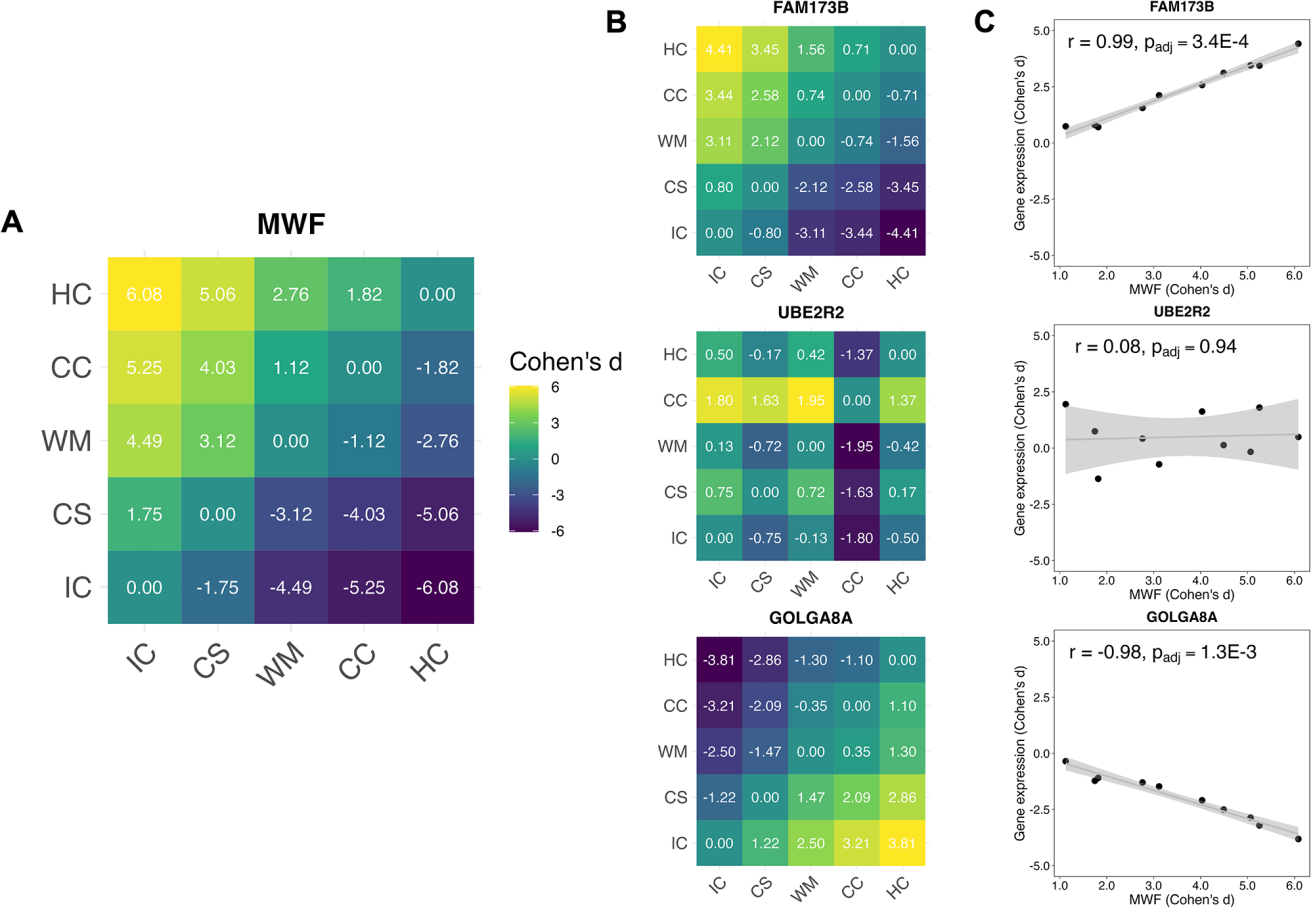
Inter-regional myelin water fraction and gene expression profiles. **(A)** Inter-regional pairwise comparison matrix for myelin water fraction (MWF), with each cell representing the Cohen’s d effect size between the five regions of interest (ROIs). **(B)** Inter-regional pairwise comparison matrices for gene expression patterns of three sample genes, FAM173B (expression has strong positive correlation with MWF), UBE2R2 (expression has no correlation with MWF), and GOLGA8A (expression has strong negative correlation with MWF), with Cohen’s d effect size between the five ROIs. **(C)** Scatterplots illustrating the correlation between MWF and gene expression for the aforementioned genes. Only data from the cells above the diagonal line of zeros in the matrices were included to eliminate redundant comparisons. Each plot includes the Pearson correlation coefficient and the FDR-adjusted p-value (p_adj_). Linear regression lines, represented in gray, are accompanied by shaded areas denoting confidence intervals. Abbreviations: IC = internal capsule posterior, CS = corticospinal tract, WM = cerebral white matter, CC = corpus callosum body, HC = hippocampus

The premise of our neuroimaging transcriptomics analysis was to compare the inter-regional expression profile of the AHBA genes against the empirical distribution pattern of MWF (Fig. 4). To achieve this, we first generated inter-regional pairwise comparison matrices for MWF (Fig. 4A) and each gene (Fig. 4B). In these matrices, each cell represents the Cohen’s d effect size between two ROIs. The Cohen’s d was chosen to enable a more representative comparability between MWF and gene expression patterns and was calculated by comparing the difference in means between two ROIs while considering the variance as given in the pooled standard deviations. We then used Pearson correlation coefficients to assess the relationship between MWF and gene expression matrices (Fig. 4C). Utilizing this methodology, we identified genes whose expression patterns mirrored the inter-regional variations observed in the MWF matrix, thereby suggesting their potential involvement in myelin-associated cell types.

### Gene categorization using single cell expression data

We leveraged the Single Cell Type Atlas provided by the Human Protein Atlas [44] to categorize the 11,074 genes that passed the two-stage consistency filter into specific cell types. This atlas presents comprehensive expression data from single-cell RNA sequencing experiments conducted on healthy human tissues and identifies genes with elevated expression in specific single cell types. Our focus was on genes exhibiting cell type-enrichment, characterized by their expression levels being at least four times higher in one cell type compared to others [45] and genes with low cell type specificity (control). We analyzed the distribution of Pearson correlation coefficients across cell types, as shown in Fig. 2. To assess statistical differences between these cell types, we utilized the Kruskal-Wallis test, which was complemented by the Mann-Whitney U test for conducting pairwise comparisons among the investigated cell types (Supplementary Table 1).

### Electronic supplementary material

Below is the link to the electronic supplementary material.

Supplementary Material 1

Supplementary Material 2

Supplementary Material 3

Supplementary Material 4

## Data Availability

The source code created for this study is available in our GitHub repository: https://github.com/kramer-lab-repo/neuroimaging_transcriptomics.git. The datasets supporting the conclusions of this article are included within the article and its additional file.

## Abbreviations

MWF: Myelin water fraction
MRI: Magnetic resonance imaging
ROIs: Regions of interest
GRASE: Gradient and spin-echo
mcDESPOT: Multicomponent driven equilibrium single pulse observation of T1 and T2
AHBA: Allen Human Brain Atlas
ANTs: Advanced Normalization Tools
IC: Internal capsule posterior
CS: Corticospinal tract
WM: Cerebral white matter
CC: Corpus callosum body
HC: Hippocampus

## References

[1] Popescu BFG, Lucchinetti CF. Pathology of demyelinating diseases. Annu Rev Pathol. 2012;7:185–217. 22313379 10.1146/annurev-pathol-011811-132443

[2] Ellison SLD, Louis DW. Greenfield’s neuropathology. 8th ed. London: CRC; 2008.

[3] MacKay AL, Vavasour IM, Rauscher A, Kolind SH, Mädler B, Moore GRW, et al. MR relaxation in multiple sclerosis. Neuroimaging Clin N Am. 2009;19(1):1–26. 19064196 10.1016/j.nic.2008.09.007

[4] Grossman RI, Gomori JM, Ramer KN, Lexa FJ, Schnall MD. Magnetization transfer: theory and clinical applications in neuroradiology. Radiogr Rev Publ Radiol Soc N Am Inc. 1994;14(2):279–90. 10.1148/radiographics.14.2.8190954 8190954

[5] Henkelman RM, Huang X, Xiang QS, Stanisz GJ, Swanson SD, Bronskill MJ. Quantitative interpretation of magnetization transfer. Magn Reson Med. 1993;29(6):759–66. 8350718 10.1002/mrm.1910290607

[6] Wolff SD, Balaban RS. Magnetization transfer contrast (MTC) and tissue water proton relaxation in vivo. Magn Reson Med. 1989;10(1):135–44. 2547135 10.1002/mrm.1910100113

[7] Beaulieu C. The basis of anisotropic water diffusion in the nervous system - a technical review. NMR Biomed. 2002;15(7–8):435–55. 12489094 10.1002/nbm.782

[8] Möller HE, Bossoni L, Connor JR, Crichton RR, Does MD, Ward RJ, et al. Iron, Myelin, and the brain: Neuroimaging meets Neurobiology. Trends Neurosci. 2019;42(6):384–401. 31047721 10.1016/j.tins.2019.03.009

[9] Weiskopf N, Mohammadi S, Lutti A, Callaghan MF. Advances in MRI-based computational neuroanatomy: from morphometry to in-vivo histology. Curr Opin Neurol. 2015;28(4):313–22. 26132532 10.1097/WCO.0000000000000222

[10] Moore GR, Leung E, MacKay AL, Vavasour IM, Whittall KP, Cover KS, et al. A pathology-MRI study of the short-T2 component in formalin-fixed multiple sclerosis brain. Neurology. 2000;55(10):1506–10. 11094105 10.1212/wnl.55.10.1506

[11] Laule C, Leung E, Lis DKB, Traboulsee AL, Paty DW, MacKay AL, et al. Myelin water imaging in multiple sclerosis: quantitative correlations with histopathology. Mult Scler Houndmills Basingstoke Engl. 2006;12(6):747–53. 10.1177/1352458506070928 17263002

[12] Laule C, Kozlowski P, Leung E, Li DKB, Mackay AL, Moore GRW. Myelin water imaging of multiple sclerosis at 7 T: correlations with histopathology. NeuroImage. 2008;40(4):1575–80. 18321730 10.1016/j.neuroimage.2007.12.008

[13] Patel Y, Shin J, Drakesmith M, Evans J, Pausova Z, Paus T. Virtual histology of multi-modal magnetic resonance imaging of cerebral cortex in young men. NeuroImage. 2020;218:116968. 32450248 10.1016/j.neuroimage.2020.116968

[14] Martins D, Giacomel A, Williams SCR, Turkheimer F, Dipasquale O, Veronese M. Imaging transcriptomics: convergent cellular, transcriptomic, and molecular neuroimaging signatures in the healthy adult human brain. Cell Rep. 2021;37(13):110173. 34965413 10.1016/j.celrep.2021.110173

[15] Liu H, Rubino C, Dvorak AV, Jarrett M, Ljungberg E, Vavasour IM, et al. Myelin Water Atlas: a template for myelin distribution in the brain. J Neuroimaging off J Am Soc Neuroimaging. 2019;29(6):699–706. 10.1111/jon.12657 31347238

[16] amethyststudio. MRI Icon, by Canva.com (2024). Retrieved from https://www.canva.com/.

[17] Becris DNA. Icon, by Canva.com (2024). Retrieved from https://www.canva.com/.

[18] Muhammad Usman. Scatter Plot Icon, by Canva.com (2024). Retrieved from https://www.canva.com/.

[19] andinur. Nerve Cell Icon, by Canva.com (2024). Retrieved from https://www.canva.com/.

[20] Adapted. from Human Brain Lobes, by BioRender.com (2024). Retrieved from https://app.biorender.com/biorender-templates.

[21] O’Brien JS, Sampson EL. Lipid composition of the normal human brain: gray matter, white matter, and myelin. J Lipid Res. 1965;6(4):537–44. 5865382

[22] Schmitt S, Cantuti Castelvetri L, Simons M. Metabolism and functions of lipids in myelin. Biochim Biophys Acta BBA - Mol Cell Biol Lipids. 2015;1851(8):999–1005. 10.1016/j.bbalip.2014.12.016 25542507

[23] Oshio K, Feinberg DA. GRASE (gradient- and spin-echo) imaging: a novel fast MRI technique. Magn Reson Med. 1991;20(2):344–9. 1775061 10.1002/mrm.1910200219

[24] Prasloski T, Rauscher A, MacKay AL, Hodgson M, Vavasour IM, Laule C, et al. Rapid whole cerebrum myelin water imaging using a 3D GRASE sequence. NeuroImage. 2012;63(1):533–9. 22776448 10.1016/j.neuroimage.2012.06.064

[25] Deoni SCL, Rutt BK, Arun T, Pierpaoli C, Jones DK. Gleaning multicomponent T1 and T2 information from steady-state imaging data. Magn Reson Med. 2008;60(6):1372–87. 19025904 10.1002/mrm.21704

[26] Deoni SCL, Matthews L, Kolind SH. One component? Two components? Three? The effect of including a nonexchanging free water component in multicomponent driven equilibrium single pulse observation of T1 and T2. Magn Reson Med. 2013;70(1):147–54. 22915316 10.1002/mrm.24429 PMC3711852

[27] Alonso-Ortiz E, Levesque IR, Pike GB. MRI-based myelin water imaging: a technical review. Magn Reson Med. 2015;73(1):70–81. 24604728 10.1002/mrm.25198

[28] West DJ, Teixeira RPAG, Wood TC, Hajnal JV, Tournier JD, Malik SJ. Inherent and unpredictable bias in multi-component DESPOT myelin water fraction estimation. NeuroImage. 2019;195:78–88. 30930311 10.1016/j.neuroimage.2019.03.049 PMC7100802

[29] Zhang J, Kolind SH, Laule C, MacKay AL. Comparison of myelin water fraction from multiecho T2 decay curve and steady-state methods. Magn Reson Med. 2015;73(1):223–32. 24515972 10.1002/mrm.25125

[30] Bouhrara M, Rejimon AC, Cortina LE, Khattar N, Bergeron CM, Ferrucci L, et al. Adult brain aging investigated using BMC-mcDESPOT-based myelin water fraction imaging. Neurobiol Aging. 2020;85:131–9. 31735379 10.1016/j.neurobiolaging.2019.10.003 PMC6924176

[31] Birkl C, Birkl-Toeglhofer AM, Endmayr V, Höftberger R, Kasprian G, Krebs C, et al. The influence of brain iron on myelin water imaging. NeuroImage. 2019;199:545–52. 31108214 10.1016/j.neuroimage.2019.05.042 PMC7610792

[32] Birkl C, Doucette J, Fan M, Hernández-Torres E, Rauscher A. Myelin water imaging depends on white matter fiber orientation in the human brain. Magn Reson Med. 2021;85(4):2221–31. 33017486 10.1002/mrm.28543 PMC7821018

[33] Ziener CH, Kampf T, Jakob PM, Bauer WR. Diffusion effects on the CPMG relaxation rate in a dipolar field. J Magn Reson San Diego Calif 1997. 2010;202(1):38–42. 10.1016/j.jmr.2009.09.016 19853483

[34] Seifert AC, Umphlett M, Hefti M, Fowkes M, Xu J. Formalin tissue fixation biases myelin-sensitive MRI. Magn Reson Med. 2019;82(4):1504–17. 31125149 10.1002/mrm.27821 PMC6626568

[35] Shatil AS, Uddin MN, Matsuda KM, Figley CR. Quantitative Ex vivo MRI changes due to Progressive Formalin fixation in whole human brain specimens: longitudinal characterization of Diffusion, Relaxometry, and myelin water fraction measurements at 3T. Front Med. 2018;5:31. 10.3389/fmed.2018.00031 PMC5826187 29515998

[36] Prasloski T, Mädler B, Xiang QS, MacKay A, Jones C. Applications of stimulated echo correction to multicomponent T2 analysis. Magn Reson Med. 2012;67(6):1803–14. 22012743 10.1002/mrm.23157

[37] Eickhoff SB, Stephan KE, Mohlberg H, Grefkes C, Fink GR, Amunts K, et al. A new SPM toolbox for combining probabilistic cytoarchitectonic maps and functional imaging data. NeuroImage. 2005;25(4):1325–35. 15850749 10.1016/j.neuroimage.2004.12.034

[38] Sunkin SM, Ng L, Lau C, Dolbeare T, Gilbert TL, Thompson CL, et al. Allen Brain Atlas: an integrated spatio-temporal portal for exploring the central nervous system. Nucleic Acids Res. 2013;41(Database issue):D996–1008. 23193282 10.1093/nar/gks1042 PMC3531093

[39] Markello RD, Arnatkeviciute A, Poline JB, Fulcher BD, Fornito A, Misic B. Standardizing workflows in imaging transcriptomics with the abagen toolbox. Jbabdi S, Makin TR, Jbabdi S, Burt J, Hawrylycz MJ, editors. eLife. 2021;10:e72129. 10.7554/eLife.72129 PMC8660024 34783653

[40] Arnatkevic̆iūtė A, Fulcher BD, Fornito A. A practical guide to linking brain-wide gene expression and neuroimaging data. NeuroImage. 2019;189:353–67. 30648605 10.1016/j.neuroimage.2019.01.011

[41] Hawrylycz MJ, Lein ES, Guillozet-Bongaarts AL, Shen EH, Ng L, Miller JA, et al. An anatomically comprehensive atlas of the adult human brain transcriptome. Nature. 2012;489(7416):391–9. 22996553 10.1038/nature11405 PMC4243026

[42] Avants BB, Tustison NJ, Song G, Cook PA, Klein A, Gee JC. A reproducible evaluation of ANTs similarity metric performance in brain image registration. NeuroImage. 2011;54(3):2033–44. 20851191 10.1016/j.neuroimage.2010.09.025 PMC3065962

[43] Fulcher BD, Little MA, Jones NS. Highly comparative time-series analysis: the empirical structure of time series and their methods. J R Soc Interface. 2013;10(83):20130048. 23554344 10.1098/rsif.2013.0048 PMC3645413

[44] Thul PJ, Lindskog C. The human protein atlas: a spatial map of the human proteome. Protein Sci Publ Protein Soc. 2018;27(1):233–44. 10.1002/pro.3307 PMC5734309 28940711

[45] Karlsson M, Zhang C, Méar L, Zhong W, Digre A, Katona B, et al. A single–cell type transcriptomics map of human tissues. Sci Adv. 2021;7(31):eabh2169. 34321199 10.1126/sciadv.abh2169 PMC8318366

