# Supplementary material for: The myelin water imaging transcriptome: myelin water fraction regionally varies with oligodendrocyte-specific gene expression
Source: Mol Brain. 2024 Jul 23;17:45. doi: 10.1186/s13041-024-01115-4 (PMC11264438; doi:10.1186/s13041-024-01115-4)
Supplement: Supplementary file 1 — Supplementary Material 1 [file 13041_2024_1115_MOESM1_ESM.docx]

**File name:** Supplementary_Table1. **File format:** .docx. **Title of data:** Pairwise comparisons of RNA single cell types. **Description of data:** Each row evaluates whether Sample 1 and Sample 2 distributions significantly differ, using the Mann-Whitney U statistic. Adjustments for multiple comparisons were made using the Bonferroni correction method. The control group encompasses genes that were classified as having low cell type specificity in the Human Protein Atlas and were present in roughly similar levels across all cell types. *: p<0.05, **: p<0.01, ***: p<0.001, ****: p<0.0001, *****: p is approximating 0.

**Supplementary Table 1. Pairwise comparisons of RNA single cell types.** Each row evaluates whether Sample 1 and Sample 2 distributions significantly differ, using the Mann-Whitney U statistic. Adjustments for multiple comparisons were made using the Bonferroni correction method. The control group encompasses genes that were classified as having low cell type specificity in the Human Protein Atlas and were present in roughly similar levels across all cell types. *: p<0.05, **: p<0.01, ***: p<0.001, ****: p<0.0001, *****: p is approximating 0.

| **Sample 1** | **Sample 2** | **Test Statistic** | **Std. Error** | **Std. Test Statistic** | **P value** | **Adjusted p value** |
| --- | --- | --- | --- | --- | --- | --- |
| Comparisons with Control | | | | | | |
| Control | Oligodendrocytes | 439.48 | 45.83 | 9.59 | 0.00  ***** | 0.00  ***** |
| Control | Excitatory neurons | -472.61 | 85.30 | -5.54 | 3.02E-8  **** | 8.45E-7  **** |
| Control | Inhibitory neurons | -350.88 | 96.55 | -3.63 | 2.79E-4  *** | 7.81E-3  ** |
| Control | Oligodendrocyte precursor cells | -152.15 | 100.15 | -1.52 | 0.13 | 1.00 |
| Control | Muller glial cells | -138.11 | 108.78 | -1.27 | 0.20 | 1.00 |
| Control | Astrocytes | -23.18 | 81.00 | -0.29 | 0.77 | 1.00 |
| Control | Adipocytes | 406.62 | 127.38 | 3.19 | 1.41E-3  ** | 3.95E-2  * |
| Comparisons with Oligodendrocytes | | | | | | |
| Oligodendrocytes | Excitatory neurons | -912.08 | 95.61 | -9.54 | 0.00  ***** | 0.00  ***** |
| Oligodendrocytes | Inhibitory neurons | -790.36 | 105.77 | -7.47 | 7.86E-14  **** | 2.20E-12  **** |
| Oligodendrocytes | Oligodendrocyte precursor cells | -591.63 | 109.06 | -5.42 | 5.80E-8  **** | 1.63E-6  **** |
| Oligodendrocytes | Muller glial cells | -577.59 | 117.03 | -4.94 | 8.00E-7  **** | 2.24E-5  **** |
| Oligodendrocytes | Astrocytes | -462.66 | 91.79 | -5.04 | 4.64E-7  **** | 1.30E-5  **** |
| Oligodendrocytes | Adipocytes | -32.86 | 134.50 | -0.24 | 0.81 | 1.00 |
| Comparisons with Excitatory Neurons | | | | | | |
| Excitatory neurons | Inhibitory neurons | -121.72 | 127.92 | -0.95 | 0.34 | 1.00 |
| Excitatory neurons | Oligodendrocyte precursor cells | -320.45 | 130.66 | -2.45 | 1.42E-2  * | 0.40 |
| Excitatory neurons | Muller glial cells | -334.49 | 137.38 | -2.43 | 1.49E-2  * | 0.42 |
| Excitatory neurons | Astrocytes | 449.42 | 116.62 | 3.85 | 1.16E-4  *** | 3.26E-3  ** |
| Excitatory neurons | Adipocytes | 879.22 | 152.53 | 5.76 | 8.21E-9  **** | 2.30E-7  **** |
| Comparisons with Inhibitory Neurons | | | | | | |
| Inhibitory neurons | Oligodendrocyte precursor cells | -198.73 | 138.26 | -1.44 | 0.15 | 1.00 |
| Inhibitory neurons | Muller glial cells | -212.77 | 144.63 | -1.47 | 0.14 | 1.00 |
| Inhibitory neurons | Astrocytes | 327.70 | 125.09 | 2.62 | 8.80E-3  ** | 0.25 |
| Inhibitory neurons | Adipocytes | 757.50 | 159.10 | 4.76 | 1.92E-6  **** | 5.39E-5  **** |
| Comparisons with Oligodendrocyte Precursor Cells | | | | | | |
| Oligodendrocyte precursor cells | Muller glial cells | 14.04 | 147.06 | 0.10 | 0.92 | 1.00 |
| Oligodendrocyte precursor cells | Astrocytes | 128.97 | 127.89 | 1.01 | 0.31 | 1.00 |
| Oligodendrocyte precursor cells | Adipocytes | 558.77 | 161.31 | 3.46 | 5.32E-4  *** | 1.49E-2  * |
| Comparisons with Muller Glial Cells | | | | | | |
| Muller glial cells | Astrocytes | 114.93 | 134.75 | 0.85 | 0.39 | 1.00 |
| Muller glial cells | Adipocytes | 544.72 | 166.80 | 3.27 | 1.09E-3  ** | 3.06E-2  * |
| Comparisons with Astrocytes | | | | | | |
| Astrocytes | Adipocytes | 429.80 | 150.17 | 2.86 | 4.21E-3  ** | 0.12 |
