# Supplementary material for: The myelin water imaging transcriptome: myelin water fraction regionally varies with oligodendrocyte-specific gene expression
Source: Mol Brain. 2024 Jul 23;17:45. doi: 10.1186/s13041-024-01115-4 (PMC11264438; doi:10.1186/s13041-024-01115-4)
Supplement: Supplementary file 2 — Supplementary Material 2 [file 13041_2024_1115_MOESM2_ESM.docx]

**File name:** Supplementary_Table2. **File format:** .docx. **Title of data:** Comparison of Pearson correlation coefficients across MWF-cell type pairings from 2 mm vs. 0 mm sample-to-region matching tolerances. **Description of data:** The median Pearson correlation coefficients [95% confidence interval] are presented.

**Supplementary Table 2. Comparison of Pearson correlation coefficients across MWF-cell type pairings from 2 mm vs. 0 mm sample-to-region matching tolerances.** The median Pearson correlation coefficients [95% confidence interval] are presented.

| **Cell Type** | **2 mm Threshold** | **0 mm Threshold** |
| --- | --- | --- |
| Oligodendrocytes | 0.71 [0.03 – 0.92] | 0.72 [0.35 – 0.91] |
| Adipocytes | 0.67 [0.24 – 0.80] | 0.75 [0.21 – 0.89] |
| Control | 0.12 [-0.71 – 0.87] | 0.48 [-0.60 – 0.93] |
| Astrocytes | 0.11 [-0.76 – 0.76] | 0.49 [-0.48 – 0.80] |
| Muller glial cells | 0.07 [-0.71 – 0.62] | 0.16 [-0.62 – 0.56] |
| Oligodendrocyte precursor cells | -0.05 [-0.60 – 0.52] | 0.17 [-0.38 – 0.47] |
| Inhibitory neurons | -0.32 [-0.83 – 0.50] | -0.53 [-0.76 – 0.44] |
| Excitatory neurons | -0.56 [-0.84 – -0.10] | -0.49 [-0.84 – 0.05] |
