# Supplementary material for: The myelin water imaging transcriptome: myelin water fraction regionally varies with oligodendrocyte-specific gene expression
Source: Mol Brain. 2024 Jul 23;17:45. doi: 10.1186/s13041-024-01115-4 (PMC11264438; doi:10.1186/s13041-024-01115-4)

**File name:** Supplementary_Figure1_2mm. **File format:** .png. **Title of data:** Microarray probe MNI coordinates using a 2 mm parcellation threshold. **Description of data:** Distribution of microarray probes included in our analysis, using a 2 mm sample-to-region matching tolerance, from five regions of interest (ROIs) across six donor brains. The ROIs and the number of microarray probes sampled from each ROI are as follows: (A) corpus callosum body (n=31), (B) corticospinal tract (n=107), (C) hippocampus (n=112), (D) internal capsule posterior (n=47), (E) cerebral white matter (n=131).


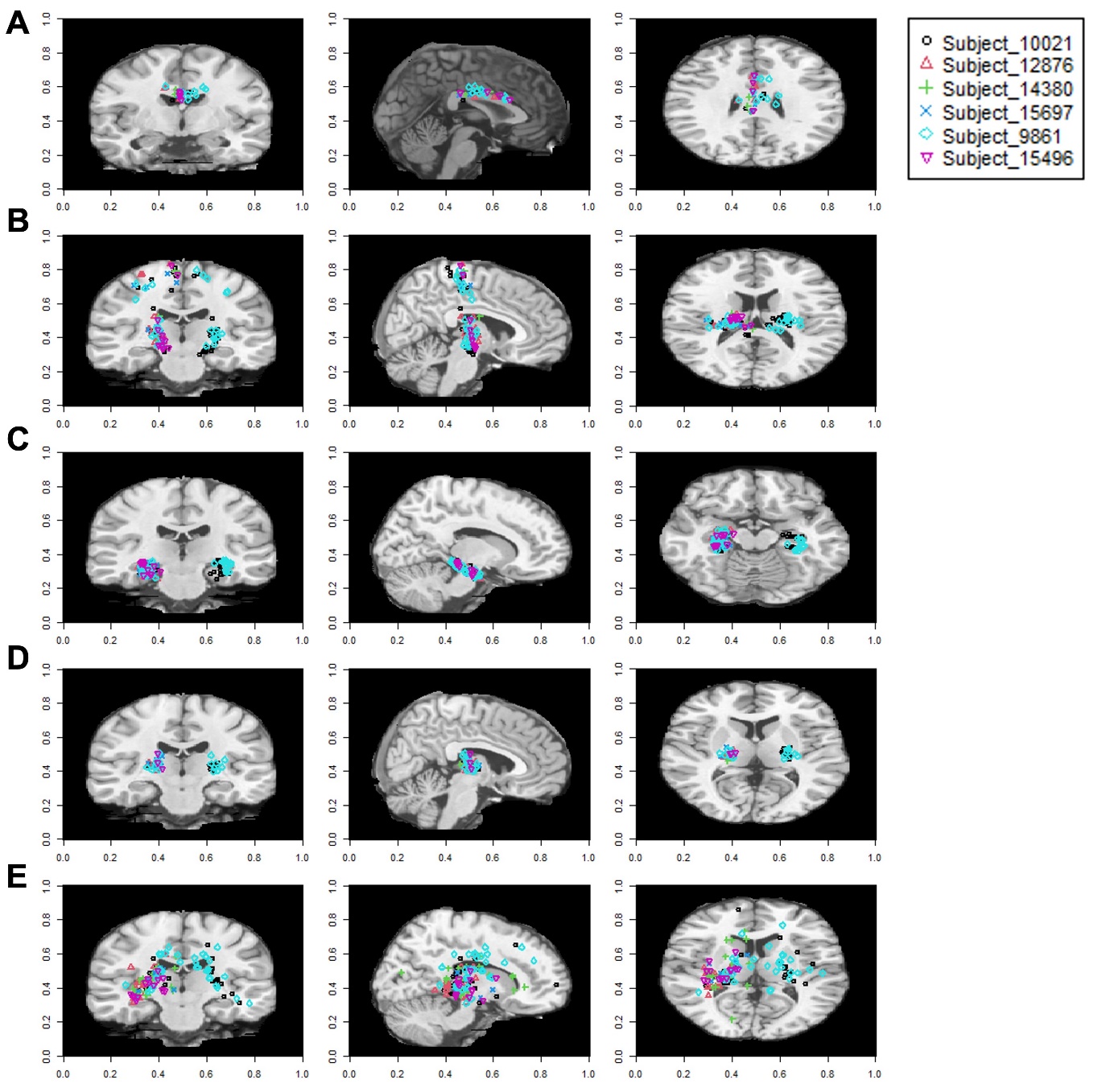

Supplement: Supplementary file 3 — Supplementary Material 3 [file 13041_2024_1115_MOESM3_ESM.docx]
